# Network analysis of neuropsychiatric symptoms in Alzheimer’s disease

**DOI:** 10.1186/s13195-023-01279-6

**Published:** 2023-08-11

**Authors:** Grace J. Goodwin, Stacey Moeller, Amy Nguyen, Jeffrey L. Cummings, Samantha E. John

**Affiliations:** 1 Department of Psychology, University of Nevada, Las Vegas (UNLV), Las Vegas, NV USA; 2 Department of Brain Health, University of Nevada, Las Vegas (UNLV), Las Vegas, NV USA; 3 Chambers-Grundy Center for Transformative Neuroscience, Department of Brain Health, University of Nevada, Las Vegas (UNLV), Las Vegas, NV USA

**Keywords:** Alzheimer’s disease, Neuropsychiatric, Network analysis, MCI, Dementia, NPI-Q, Agitation, Disinhibition, Depression

## Abstract

**Background:**

Neuropsychiatric symptoms due to Alzheimer’s disease (AD) and mild cognitive impairment (MCI) can decrease quality of life for patients and increase caregiver burden. Better characterization of neuropsychiatric symptoms and methods of analysis are needed to identify effective treatment targets. The current investigation leveraged the National Alzheimer’s Coordinating Center (NACC) Uniform Data Set (UDS) to examine the network structure of neuropsychiatric symptoms among symptomatic older adults with cognitive impairment.

**Methods:**

The network relationships of behavioral symptoms were estimated from Neuropsychiatric Inventory Questionnaire (NPI-Q) data acquired from 12,494 older adults with MCI and AD during their initial visit. Network analysis provides insight into the relationships among sets of symptoms and allows calculation of the strengths of the relationships. Nodes represented individual NPI-Q symptoms and edges represented the pairwise dependency between symptoms. Node centrality was calculated to determine the relative importance of each symptom in the network.

**Results:**

The analysis showed patterns of connectivity among the symptoms of the NPI-Q. The network (*M* = .28) consisted of mostly positive edges. The strongest edges connected nodes within symptom domain. Disinhibition and agitation/aggression were the most central symptoms in the network. Depression/dysphoria was the most frequently endorsed symptom, but it was not central in the network.

**Conclusions:**

Neuropsychiatric symptoms in MCI and AD are highly comorbid and mutually reinforcing. The presence of disinhibition and agitation/aggression yielded a higher probability of additional neuropsychiatric symptoms. Interventions targeting these symptoms may lead to greater neuropsychiatric symptom improvement overall. Future work will compare neuropsychiatric symptom networks across dementia etiologies, informant relationships, and ethnic/racial groups, and will explore the utility of network analysis as a means of interrogating treatment effects.

**Supplementary Information:**

The online version contains supplementary material available at 10.1186/s13195-023-01279-6.

Alzheimer’s disease (AD) is the most common cause of dementia in older adults. As of 2022, it was estimated that 6.6 million adults aged 65 and older in the United States were living with AD [1]. This number is expected to grow to a projected 12.7 million people by 2050. AD is characterized by insidious onset of amnestic symptoms, followed by deterioration of other cognitive abilities and functional independence [2]. Patients with mild cognitive impairment (MCI) and AD additionally exhibit behavioral or neuropsychiatric changes.

Neuropsychiatric symptoms (NPS) refer to behavioral, affective, and personality changes that can be attributed to underlying neurodegenerative disease. Common symptoms include apathy, depression, aggression, anxiety, and sleep disturbance, and less common symptoms include irritability, appetite changes, aberrant motor behavior, delusions, disinhibition, euphoria, and hallucinations [3]. Almost all patients exhibit neuropsychiatric symptoms at some point in their disease [4–6], and apathy and depression are the most frequently reported disturbances among patients with AD [4, 7] and MCI [5, 8]. Neuropsychiatric symptoms are often present in the early clinical stages of neurocognitive decline and are therefore considered diagnostic and prognostic indicators of neurodegenerative disease [9–14]. The type of symptoms expressed may also indicate underlying pathological changes. Previous work has highlighted the influence of neurofibrillary tau burden on the presence of agitation, anxiety, appetite change, depression, and sleep disturbance [15]. A recent review highlighted the neuroanatomical correlates of NPS in AD and identified symptom-general (e.g., anterior cingulate cortex and orbitofrontal cortex) and symptom-specific patterns (e.g., frontal-limbic circuit involvement in depression) [16].

Neuropsychiatric symptoms are distressing for patients and caregivers and are associated with increased functional [8] and cognitive impairment [3, 17–20], hospitalization, caregiver burden [21], and institutionalization [4, 22]. Nonpharmacologic interventions (e.g., environmental modifications, exercise, reminiscence therapies, caregiver training) are considered the first line of management of neuropsychiatric symptoms. However, most patients are eventually treated with psychotropic medications as the disease progresses and symptoms worsen. Evidence of efficacy of nonpharmacologic and pharmacologic interventions is mixed; while some patients and caregivers experience relief from treatment, others do not [23].

Assessment of neuropsychiatric symptoms in AD is important for accurate differential diagnosis, disease management, and understanding the neurobiological underpinnings of behavioral changes in dementia [24]. The Neuropsychiatric Inventory Questionnaire (NPI-Q) is a widely used informant-based questionnaire that assesses the presence and severity of 12 neuropsychiatric symptoms evident within the last month [25]. Previous studies have used factor analysis, cluster analysis, and latent class analysis to categorize symptoms of the NPI and NPI-Q; however, the taxonomy of neuropsychiatric symptoms in AD remains unclear. There is relatively low concordance among studies attempting to identify neuropsychiatric symptom clusters or domains [26]. Some studies have identified 3 symptom domains [27–29] while others have identified 4 or more [13, 19, 30]. Importantly, these item-level and domain-level examinations do not capture symptom complexity, interaction, or comorbidity. One study examining comorbidity among neuropsychiatric symptoms among patients with AD identified several statistically significant combinations of symptoms (e.g., hallucinations were 6.49 times higher in those with delusions) [29]; however, this approach does not consider the co-occurrence of multiple (i.e., more than two) neuropsychiatric symptoms [31].

Recently, researchers have posited that network models could provide a detailed characterization of psychological syndromes [32]. According to network theory, psychological disorders can be viewed as a set of interacting symptoms that amplify, reinforce, and maintain each other [33–35]. Network analysis highlights clusters of strongly interconnected symptoms and quantifies the relative importance of individual symptoms [36, 37]. Central symptoms, or symptoms with a large number of connections to other symptoms in a network, represent core features of a syndrome [35], and can, theoretically, be considered targets for widespread symptom reduction [38]. Network analysis has been used to characterize symptom presentation and progression in schizophrenia [39], depression, anxiety [40], post-traumatic stress disorder [41], and sport-related concussion [42–44]. Three studies have used network analysis to characterize NPS within clinical memory samples [8, 31, 45], evaluating frequent and central symptoms [31], longitudinal stability of clinical presentation [45], and the relationship of NPS to adaptive functioning in AD [8]. None of these have characterized the network structure of the 12-item NPI-Q, which may illuminate patterns of comorbidity in AD. Network analysis could provide unique insights into symptom maintenance and progression and identify central symptoms that may be efficient targets for widespread symptom reduction.

The current investigation leveraged the National Alzheimer’s Coordinating Center (NACC) Uniform Data Set (UDS) to examine the network structure of neuropsychiatric symptoms among older adults with cognitive impairment. Given that the presence and nature of initial symptoms consistently predict disease course, data from participants’ initial visit were used. We examined network structure among symptomatic patients diagnosed with MCI or dementia due to AD. This study aimed to conceptualize the comorbidity and complexity of neuropsychiatric symptoms in AD by examining a binary network (e.g., symptoms present or absent) and provide a foundation for personalized approaches to symptom management.

## Method

The NACC UDS is a comprehensive data repository for research on neurodegenerative disorders, including AD. The UDS contains longitudinal data that have been collected since 2005 at National Institute on Aging (NIA)-funded Alzheimer’s Disease Research Centers (ADRCs) across the United States [46, 47]. Data elements and collection methods have been described previously [48–51]. The NACC UDS includes neuropsychological, behavioral, medical, and health history data that is used to accurately diagnose neurodegenerative disease and track its course [51]. Participants and study partners enrolled at each ADRC provide written consent as part of the IRB-approved protocol at that site. This consent covers both the data collection procedures required by the respective center as well as the inclusion of the participant’s data in the larger NACC UDS database.

### Participants

**Fig. 1:**
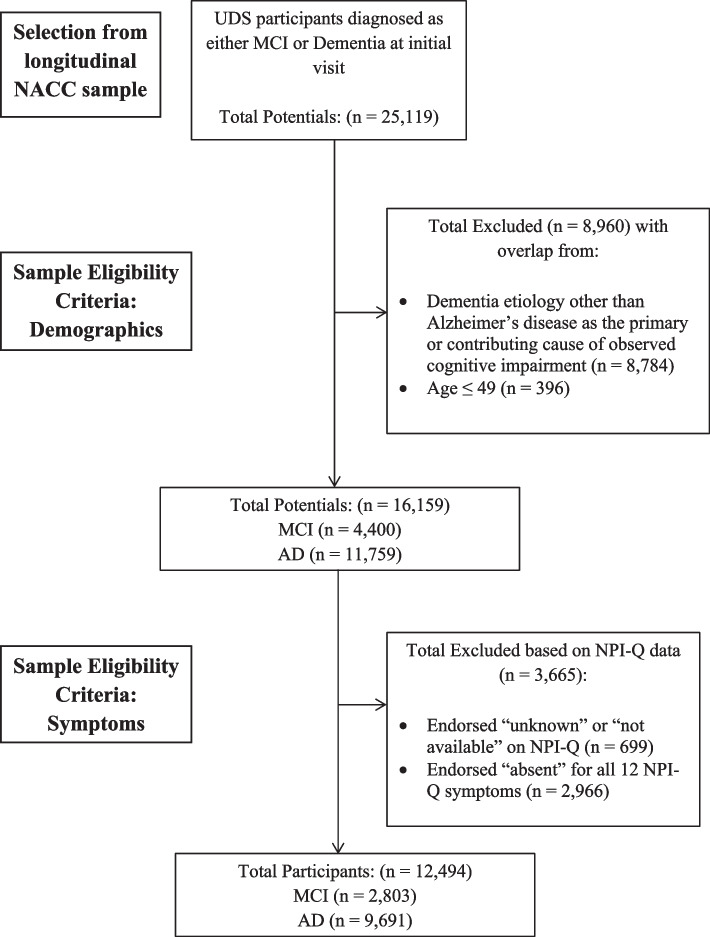
Participant selection diagram

Participants were selected from the NACC UDS (v1-v3) data set (https://naccdata.org/). Participant evaluations from initial visits were used in the current analysis and were completed at funded ADRCs during the period between September 2005 and the freeze date of December 2021. Patient demographic variables and diagnostic status were used to identify the sample for analysis (Fig. 1). The total sample for all initial participant visits was 44,713. The following inclusion criteria were applied for sample identification: cognitive status of MCI or dementia (*n* = 25,119); AD was the primary or contributing cause of observed impairment (*n* = 16,335); participants were 50 years or older (*n* = 16,159); and at least one symptom on the NPI-Q was endorsed. Participants were excluded if they endorsed “unknown” or “not available” on any NPI-Q items. The final sample (*n* = 12,494) consisted of older adults (*M*_age_ = 73.9, *SD*_age_ = 9.37; 46.2% male, 53.8% female, *M*_education_ = 15.21 years, *SD*_education_ = 8.58 years) who predominantly identified as non-Hispanic white (74.5% non-Hispanic white, 11% non-Hispanic Black, 8.5% other, 5.8% Hispanic white, 0.3% Hispanic Black). The majority of the sample met criteria for dementia (77.6% dementia, 22.4% MCI) and AD was the presumed primary etiology in 93.9% and contributing etiology in 6.1%. See Table 1 for demographic and descriptive data.

**Table 1:** Participant demographics stratified by cognitive status

Participant Demographics Stratified by Cognitive Status (*N* = 12,494)		
	MCI	Dementia
*n*	2803	9691
Age [M(*SD*)]	73(*8.23*)	73.9(*9.56*)
Sex		
Male	50.48%	44.98%
Female	49.52%	55.02%
Education Years [M(*SD*)]	16(*6.81*)	15(*9.01*)
Ethnic Racial Group (%)		
non-Hispanic white	75.10%	74.26%
non-Hispanic Black	10.49%	11.09%
Hispanic white	5.42%	5.91%
Hispanic Black	0.32%	0.24%
Other	8.67%	8.49%
Alzheimer's Disease Etiology		
Primary Etiology	94.18%	93.81%
Contributing Etiology	5.82%	6.19%
CDR Global Impairment rating (%)		
None (0.0)	3.28%	0.29%
Questionable (0.5)	94.40%	28.99%
Mild (1.0)	2.32%	45.73%
Moderate (2.0)	0.00%	17.19%
Severe (3.0)	0.00%	7.80%
GDS Total Score[M(*SD*)]	2.64(*2.57*)	2.73(*2.72*)

*M* mean, *SD* standard deviation, *MCI* mild cognitive impairment, *CDR* clinical dementia rating, *GDS* geriatric depression scale, MCI vs. Dementia derived from Cognitive Status at UDS Visit variable. Alzheimer’s disease etiology derived from clinician diagnosis of cause of observed cognitive impairment due to Alzheimer’s disease. Impairment ratings derived from the Clinical Dementia Rating Global Impairment score. Depression derived from Geriatric Depression Scale total score

### Measures

#### Race and ethnicity

In order to examine participant race and ethnicity, a new variable was calculated that combined data from the NACC-derived race variable for the six main census race groups and the UDS ethnicity variable for Hispanic/Latino ethnicity. Five new racial/ethnic groups were created from these data: non-Hispanic white, Hispanic white, Non-Hispanic Black, Hispanic Black, and all other categories.

#### *Cognitive status and Alzheimer’s disease statu*s

Cognitive status and etiologic diagnosis for each patient was determined through a formal process at each ADRC using the 2011 National Institute on Aging-Alzheimer’s Association (NIA-AA) guidelines [46, 50]. Diagnoses are assigned by either a consensus panel of experts or by the single physician conducting the examination, and this varies by center. Cognitive status includes the following categories: 1) normal cognition, 2) impaired-not-MCI (subjects who are cognitively impaired according to neuropsychological performance but who do not meet NIA-AA criteria for MCI), 3) MCI (subjects with either amnestic or non-amnestic MCI), and 4) dementia (subjects who have a cognitive diagnosis of dementia) [52]. AD etiology includes the following categories: 1) primary (AD is the primary cause of observed cognitive impairment), 2) contributing (AD is a contributing cause of observed cognitive impairment), 3) non-contributing (AD was a non-contributing cause of observed cognitive impairment), 4) cognitively impaired but not AD (no etiological diagnosis of AD), and 5) diagnosis of normal cognition [51]. Only those with a cognitive diagnosis of MCI or dementia and those with an etiology of AD as a primary or contributing cause of observed impairment were included in the analysis sample.

#### Characterization variables

The Geriatric Depression Scale (GDS) is a self-report measure of depression symptoms [53]. Patients rate whether or not they experienced 15 depression symptoms over the last week (0 = *No*, 1 = *Yes*). Scores are summed and scores of 9–11 indicate moderate depression and scores of 12–15 indicate severe depression. The Clinical Dementia Rating (CDR) ® Dementia Staging Instrument is a 5-point scale that characterizes six domains of cognitive and functional abilities [54]. Information is obtained through semi-structured interview of the patient and informant, and clinicians rate the patient’s level of overall impairment (0.0 = *No impairment*–3.0 = *Severe Impairment*).

#### Primary outcome measure

The NPI-Q is a widely used measure to assess neuropsychiatric symptoms among clinical populations [25]. The NPI-Q relies on a caregiver/informant report of the presence, severity, and distress caused by 12 neuropsychiatric symptoms evident within the past month. Assessed symptoms include delusions, hallucinations, agitation/aggression, depression/dysphoria, anxiety, elation/euphoria, apathy/indifference, disinhibition, irritability/lability, motor disturbance, nighttime behaviors, and appetite/eating problems [25]. Severity of each symptom is rated on a three-point scale (1 = *Mild*, 2 = *Moderate*, 3 = *Severe*) and Caregiver Distress is rated on a six-point scale (0 = *Not distressing at all*, 1 = *Minimal*, 2 = *Mild*, 3 = *Moderate*, 4 = *Severe*, 5 = *Extreme*). For use within the present analyses, our primary outcome measure was the absence or presence of each symptom (0 = *No*, 1 = *Yes*) [25]. The overall endorsement score ranges from 0 to 12. The NPI-Q has adequate psychometric properties, including acceptable test–retest reliability and convergent validity [25].

### Analyses

#### Network estimation

Statistical analyses were conducted in *R* version 4.0.3. using *qgraph *[55],* bootnet *[56], and *networktools *[57]. Network analysis allows for the graphical representation of symptoms (nodes) and the statistical relationship among them (edges). Item endorsement on the NPI-Q is dichotomous (i.e., symptoms are either present or absent), so methods that calculate partial correlations between nodes are not appropriate for analysis, given that they require assumptions of linearity and normality [58]. Instead, a binary equivalent of the Gaussian approximation method was used. The *eLasso *method, which is based on the Ising model, estimates parameters using logistic regressions [58].

The network was estimated from individual NPI-Q item scores. Nodes represent the threshold of each NPI-Q symptom, or the independent disposition of that symptom to be present or absent without the influence of neighboring symptoms. Each node is regressed on all other nodes in the network. Edges represent the pairwise dependency between two nodes after controlling for all other nodes in the network. The network represents the conditional probability of an observed binary variable (e.g., presence/absence of delusions) given all other measured variables (e.g., presence/absence of all other NPI-Q symptoms) [58, 59].

Two methods were applied to balance network sensitivity and specificity. First, networks were regularized using the recommended least absolute shrinkage and selection operator (LASSO) penalty [58]. The tuning parameter was chosen using the Extended Bayesian Information Criterion (EBIC) [60]. The EBIC hyperparameter gamma value was set to 0.25, which is recommended for estimating binary networks [58]. This process removes weak and spurious edges and returns a sparse network in which a small number of likely genuine edges are used to explain network structure [59]. Second, the “OR-rule” was used to determine the final set of edges. The “OR-rule” requires only one of the two regression coefficients to be nonzero (i.e., for nodes *j* and *k,* either *b*_*jk*_* or b*_*kj*_ is nonzero) in order for the edge to be retained in the network, thereby increasing the number of estimated connections. Alternatively, a stricter “AND-rule” can be applied, which requires *both *regression coefficients to be nonzero for the edge to be retained in the network [58]. The less stringent “OR-rule” was more appropriate in this study given that regularization had already been applied.

Once the final edges were selected, the weighted value of each edge was calculated by taking the mean of both regression coefficients (i.e., for nodes *j* and *k,* the average of *b*_*jk*_ and *b*_*kj*_) for a given pair of nodes. The final network consisted of weighted edges between all node pairs and represented a statistical association between nodes after controlling for all other nodes in the network [58]. The Fruchterman-Reingold algorithm was used for the graph layout, such that nodes were placed close together if they had stronger or more connections to each other [58, 61].

#### Node centrality

Centrality was computed to determine symptoms’ relative importance within the network. Node *strength* and *expected influence* measure the number of connections extending from a given node that is weighted by *eLasso *coefficients [37, 58, 62]. *Strength *is calculated by taking the sum of the absolute value of all edges extending from a given node [37]. *Expected influence *considers negative edges and is calculated by taking the sum of all edges extending from a given node [58]. For both metrics, higher values indicate greater node importance [37, 62].

#### Network accuracy

Edge-weight accuracy, centrality stability, and edge-weight and centrality difference tests were computed to determine network accuracy [37]. To measure edge-weight accuracy, nonparametric bootstrapped confidence intervals (CIs, 95%) were constructed around the regularized edge-weights. Large CIs suggest that edge-weights do not significantly differ. To assess centrality stability, a case-dropping subset bootstrap approach was employed. The centrality stability (*CS*) coefficient signifies the maximum proportion of cases that can be dropped while maintaining a large correlation (*r* = .70) between the full- and subset-sample networks’ centrality values. *CS-*coefficients should be above .50 and no lower than .25 for the centrality indices to be trustworthy [37]. Edge-weight and node centrality differences were examined using calculated difference scores for each pair of bootstrapped edge-weight/centrality. Edge-weights and centralities are considered trustworthy if zero is included in the bootstrapped CI.

## Results

**Fig. 2:**
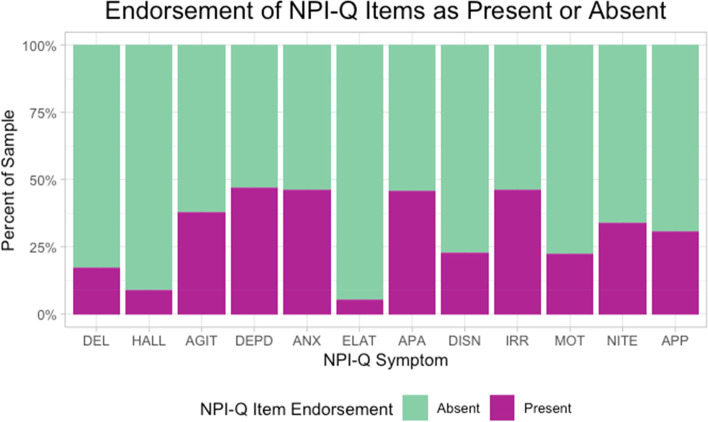
Endorsement of NPI-Q Items as present or absent. Note. Percent of sample with presence or absence of individual NPI-Q symptoms based on informant report. Symptom present = 1 or NPI-Q item endorsed. Symptom absent = 0 or NPI-Q item not endorsed. “DEL” = Delusions, “HALL” = hallucinations, “AGIT” = agitation/aggression, “DEPD” = depression/dysphoria, “ANX” = anxiety, “ELAT” = elation/euphoria, “APA” = apathy/indifference, “DISN” = disinhibition, “IRR” = irritability/lability, “MOT” = motor disturbance”, “NITE” nighttime behaviors, “APP” appetite/eating problems

On average, 3 or more symptoms were endorsed on the NPI-Q (MCI: *M* = 2.75, *SD* = 1.82, range = 1–12; dementia: *M* = 3.90, *SD* = 2.32, range = 1–12). Symptom severity was mild overall (MCI: *M* = 3.78, *SD* = 3.32; dementia: *M* = 6.05, *SD* = 4.78)*.* The most frequently endorsed symptom was depression/dysphoria (46.9%), followed closely by irritability/lability (46.2%), anxiety (46.1%), and apathy/indifference (45.8%) (Fig. 2)*.* See Table 1 for additional sample characterization through summary of CDR scores.

### Network architecture

**Fig. 3:**
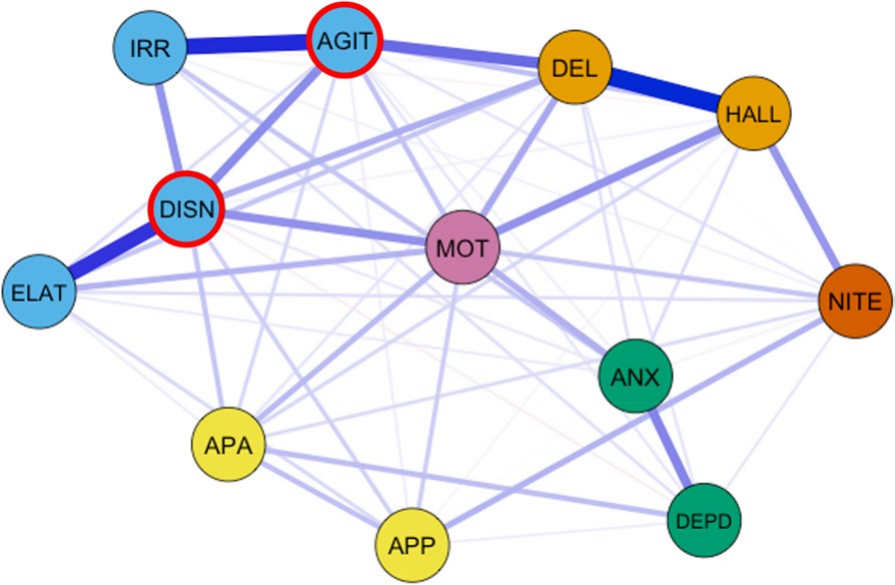
Network of neuropsychiatric symptoms. Note. The layout of the graph used the Fruchterman-Reingold algorithm. Colors were added manually to highlight statistically derived clusters. Nodes with highest strength centrality and expected influence are outlined in red. “DEL” = Delusions, “HALL” = hallucinations, “AGIT” = agitation/aggression, “DEPD” = depression/dysphoria, “ANX” = anxiety, “ELAT” = elation/euphoria, “APA” = apathy/indifference, “DISN” = disinhibition, “IRR” = irritability/lability, “MOT” = motor disturbance”, “NITE” nighttime behaviors, “APP” appetite/eating problems

Out of a possible 66 edges, 57 (86%) were retained (*M*_weight_ = .28) following regularization. The network consisted of mostly positive edges (Fig. 3). Four symptom clusters were identified visually using the Fruchterman-Reingold algorithm: 1) irritability/lability, agitation/aggression, disinhibition and elation/euphoria; 2) delusions and hallucinations; 3) anxiety and depression/dysphoria; 4) apathy/indifference and appetite/eating problems. The strongest edges within the same symptom clusters were found between delusions and hallucinations (edge-weight = 1.51), agitation/aggression and irritability/lability (edge-weight = 1.31), elation/euphoria and disinhibition (edge-weight = 1.21), depression/dysphoria and anxiety (edge-weight = .72), agitation/aggression and disinhibition (edge-weight = .68), and disinhibition and irritability/lability (edge-weight = .63). The network also connected nodes from different symptom clusters: agitation/aggression (edge-weight = .83), disinhibition and motor disturbance (edge-weight = .65), hallucinations and motor disturbance (edge-weight = .64), and hallucinations and nighttime behaviors (edge-weight = .61).

**Fig. 4:**
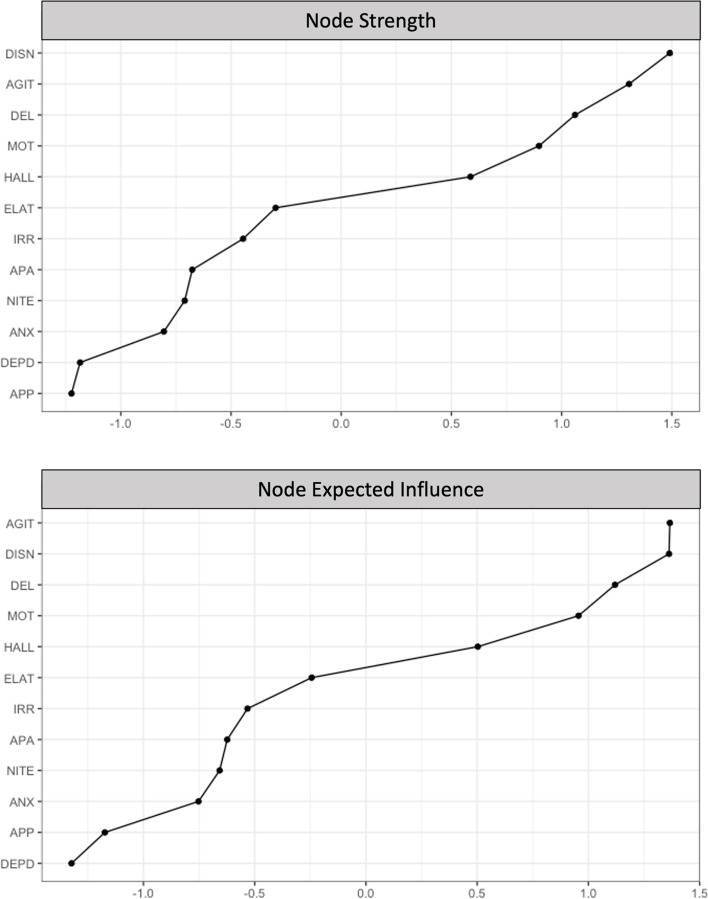
Rank order of node strength and expected influence values. Note. Rank order of node strength (top graph) and expected influence (bottom graph). Nodes are presented in order from highest (top of figure) to lowest strength (bottom of figure). Expected influence values are shown as standardized *z-*scores

Node strength (*CS*(cor = .7) = .75) and expected influence (*CS*(cor = .7) = .75) were stable and are interpretable indices of centrality (Supplementary Fig. 1). Disinhibition had the highest node strength (*z* = 1.49), and agitation/aggression had the highest expected influence (*z* = 1.37). Disinhibition and agitation/aggression shared most of their connections with other behavioral symptoms, including irritability/lability, elation/euphoria, and motor disturbance. Depression/dysphoria and appetite/eating problems had the lowest node strength and expected influence (Fig. 4).

#### Network accuracy

Confidence intervals were wider than optimal around the parameter estimates for edge-weight, suggesting that estimation of edge-weight values should be interpreted with caution (Supplementary Fig. 2). While there were considerable overlaps among the edge-weight CIs, there was no overlap around the strongest edges in the network, suggesting that the order of the strongest edges are accurate and interpretable.

Bootstrapped differences tests showed that edge-weight values significantly differed from one another, providing additional evidence that the order of edge-weight values is interpretable (Supplementary Fig. 3). Additionally, node centrality values significantly differed from one another, providing additional evidence that the order of centrality values is interpretable (Supplementary Figs. 4 and 5). In sum, results suggest that the network was accurate, stable, and interpretable.

## Discussion

The present study used network analysis to examine the associations among neuropsychiatric symptoms occurring in a large sample of symptomatic older adults with cognitive impairment. Neuropsychiatric symptoms become increasingly evident throughout AD progression and are the most likely symptoms to require behavioral and pharmacological intervention [63]. These symptoms, along with other behavioral symptoms, are difficult to manage, are highly distressing, and confer risk for patients, caregivers, and clinicians [64]. Moreover, these behaviors are often directed toward or experienced by caregivers, which leads to increased caregiver burden and decreased quality of life [63, 65].

Within our analytic sample, participants had mostly mild (MCI) or moderate (dementia) global impairment. Three or more neuropsychiatric symptoms were endorsed on average, and neuropsychiatric and depression symptom severity were mild overall. Consistent with previous research [29], the interconnectedness of symptoms observed in the network suggests that neuropsychiatric symptoms are highly comorbid. Using the Fruchterman-Reingold algorithm, four symptom clusters were identified, encompassing 10 of the 12 symptoms and illustrating patterns observed in previous research. Symptom clusters in the present analyses highlight common clinical patterns observed across disorders, including the cluster with anxiety and depression and the cluster of delusions and hallucinations [15, 16]. The largest symptom cluster, composed of irritability/lability, agitation/aggression, disinhibition, and elation/euphoria mirrors anatomic studies highlighting frontal-subcortical involvement in AD [66] and supports previous research on impulsive dyscontrol symptoms in MCI and subjective cognitive decline [31]. Two symptoms, motor disturbance and nighttime behaviors, were not included in any of the clusters; previous research has noted the uncommon endorsement of motor disturbance within AD samples [8].

While the present model cannot determine causality, results suggest that neuropsychiatric symptoms may be mutually reinforcing, whereby activation of one symptom results in cascading activation of other symptoms throughout the network. For example, disinhibition was associated with motor disturbance, motor disturbance was associated with hallucinations, hallucinations were associated with nighttime behaviors, and nighttime behaviors were associated with appetite and eating problems.

As in previous studies [5, 67], depression was the most commonly endorsed symptom in the current sample. However, depression was not a highly central symptom in the network. Our results suggest that depression, while common, is not predictive of neuropsychiatric symptoms more broadly. However, given that depression in AD is associated with greater functional and cognitive disability, caregiver burden, and reduced quality of life [67], it may be an important standalone symptom to evaluate and ameliorate in this population.

Disinhibition and agitation/aggression emerged as central symptoms in the network, suggesting that they likely influence the activation or persistence of other neuropsychiatric symptoms. Disinhibition refers to difficulty suppressing inappropriate or maladaptive thoughts or behaviors [68]. Agitation is characterized by physical aggression, verbal aggression, resistance to attempts at care, and hyperactivity. Aggression refers to more marked verbal insults (e.g., shouting, cursing) and physical behaviors (e.g., hitting, kicking, biting, throwing objects). With respect to symptom aggregation, the presence of disinhibition increases the likelihood of all other behavioral symptoms being present and is most strongly linked to agitation. Symptoms with strong relationships to one another within the network, as with irritability and agitation, may reflect strong temporal associations and co-occurrence, as irritability is often a precursor or accompanying feature of agitation/aggression [69]. Thus, when agitation is present and endorsed, irritability is likely also to have occurred. Although speculative, our observations suggest that the presence of some neuropsychiatric symptoms predicts other neuropsychiatric symptoms.

According to network theory of mental disorders, central symptoms represent core features of a syndrome, and “deactivating” a core symptom could, in turn, deactivate other symptoms within the network [33]. Thus, treating or managing disinhibition and agitation/aggression may predict alleviation of overall neuropsychiatric symptoms. In sum, our findings lend further support to the importance of these network relationships as key features of neuropsychiatric symptoms in AD.

### Limitations and future directions

While this study provides initial information about neuropsychiatric symptom comorbidity, the use of dichotomous variables results in a loss of valuable information. Networks that include symptom frequency, severity, and degree of symptom burden would provide more nuanced information about the interconnectedness of the 12 neuropsychiatric symptoms. Given the cross-sectional design of this study, we cannot infer temporal precedence between symptoms. It is important that future research continues to explore patterns across the disease course among neuropsychiatric symptoms to better identify conversion risk and determine whether neuropsychiatric symptom networks change as disease progresses. Additionally, while central symptoms can be considered theoretical targets for reducing associations among other symptoms, treatment simulation studies are mixed [70] and empirical data are needed. This work should be replicated in NPI-Q networks of patients before and after intervention to determine the extent to which other neuropsychiatric symptoms are reduced when central symptoms are removed or ameliorated.

The NPI-Q is an informant-based measure and symptoms can be misinterpreted, underreported, or overreported. Further, the NPI-Q asks informants to endorse symptoms only if they have occurred in the past month, which does not consider fluctuating disease presentations. Network relationships should be studied using patient or clinician reports to determine if network structure persists across different informant relationships (e.g., spousal caregivers vs. siblings vs. children) and characteristics (e.g., time spent with participant and/or residential setting). NPI-Q symptom descriptions may be subject to cultural bias wherein the informant does not acknowledge or interpret the symptom as part of the disease. Relatedly, ethnic and racial differences in neuropsychiatric symptomatology remain understudied and should be addressed in future work. While our analyses incorporated data from a diverse ethnic and racial cohort, future analyses will examine these relationships more intentionally. Finally, examining the extent to which pre-morbid, environmental, and sociodemographic factors may moderate the interrelationships among neuropsychiatric symptoms could better characterize symptom heterogeneity. Areas for future research may center on associations of neuropsychiatric symptom clusters with other markers of disease, such as apolipoprotein E genotype, cerebrospinal fluid biomarkers, and amyloid and tau positron emission tomography.

## Conclusions

In summary, this study examined the network structure of neuropsychiatric symptoms occurring among older adults with MCI and AD dementia. Results quantify the relationships between symptom pairs and identify highly influential symptoms in the network. Our findings highlight neuropsychiatric symptom comorbidity and suggest that disinhibition and agitation/aggression may be important targets for intervention. A network perspective may improve current understanding of neuropsychiatric symptomatology in this population. Future research is needed to determine the clinical utility of network models in assessment and treatment.

### Supplementary Information

**Additional file 1: Supplementary Figure 1.** Average Correlations Between Node Strength and Expected Influence of Original Network and Networks Sampled With Persons Dropped. **Supplementary Figure 2.** Bootstrapped Confidence Intervals of Estimated Edge-Weights. **Supplementary Figure 3.** Bootstrapped Difference Tests Between Edge-Weights That Were Non-Zero in the Estimated Network. **Supplementary Figure 4.** Bootstrapped Difference Tests Between Node Strength in the Estimated Network. **Supplementary Figure 5.** Bootstrapped Difference Tests Between Node Expected Influence in the Estimated Network.

## Data Availability

The dataset used and analyzed in the current study are available through the NACC data request process. Code can be obtained from the corresponding author upon request.

## Abbreviations

AD: Alzheimer’s disease
MCI: Mild cognitive impairment
NPS: Neuropsychiatric symptoms
NPI-Q: Neuropsychiatric Inventory Questionnaire
NPI: Neuropsychiatric Inventory
NACC: National Alzheimer’s Coordinating Center
UDS: Uniform Data Set
NIA: National Institute on Aging
ADRC: Alzheimer’s Disease Research Center
IADL: Instrumental activities of daily living
GDS: Geriatric Depression Scale
CDR: Clinical Dementia Rating
EBIC: Extended Bayesian information criterion
CI: Confidence interval
CS: Centrality stability
