# Supplementary material for: Network analysis of neuropsychiatric symptoms in Alzheimer’s disease
Source: Alzheimers Res Ther. 2023 Aug 11;15:135. doi: 10.1186/s13195-023-01279-6 (PMC10416506; doi:10.1186/s13195-023-01279-6)
Supplement: Supplementary file 1 — Additional file 1: Supplementary Figure 1. Average Correlations Between Node Strength and Expected Influence of Original Network and Networks Sampled With Persons Dropped. Supplementary Figure 2. Bootstrapped Confidence Intervals of Estimated Edge-Weights. Supplementary Figure 3. Bootstrapped Difference Tests Between Edge-Weights That Were Non-Zero in the Estimated Network. Supplementary Figure 4. Bootstrapped Difference Tests Between Node Strength in the Estimated Network. Supplementary Figure 5. Bootstrapped Difference Tests Between Node Expected Influence in the Estimated Network. [file 13195_2023_1279_MOESM1_ESM.docx]

**Supplementary Material**

Supplementary Figure 1. Average Correlations Between Node Strength and Expected Influence of Original Network and Networks Sampled With Persons Dropped


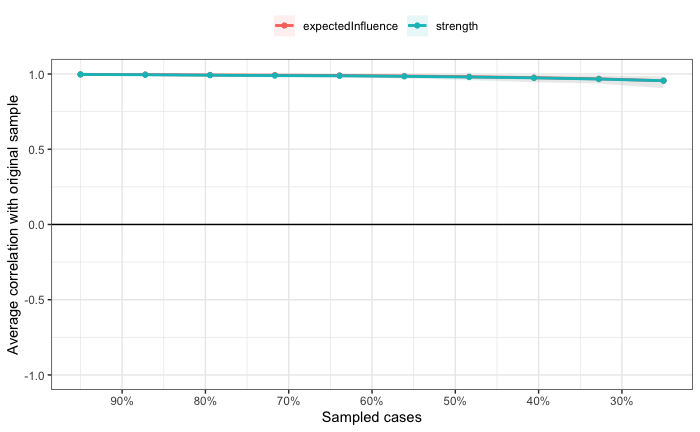


Supplementary Figure 2. Bootstrapped Confidence Intervals of Estimated Edge-Weights.


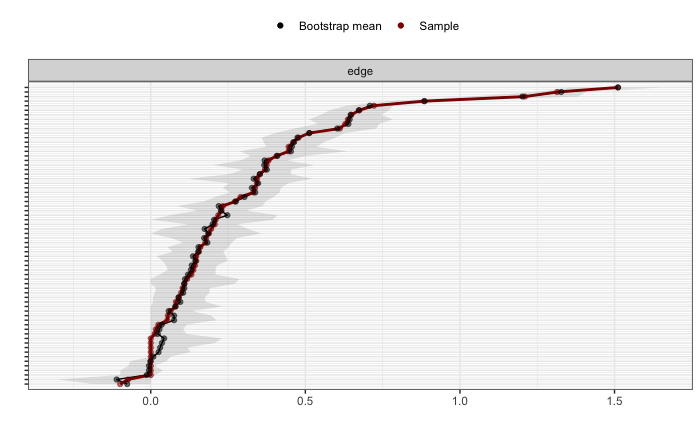


*Note.* Red lines represents sample values. Gray lines represent bootstrapped confidence intervals. Each horizontal line represents one edge from the network. Edges are ordered from lowest to highest edge-weight.

Supplementary Figure 3. Bootstrapped Difference Tests Between Edge-Weights That Were Non-Zero in the Estimated Network.


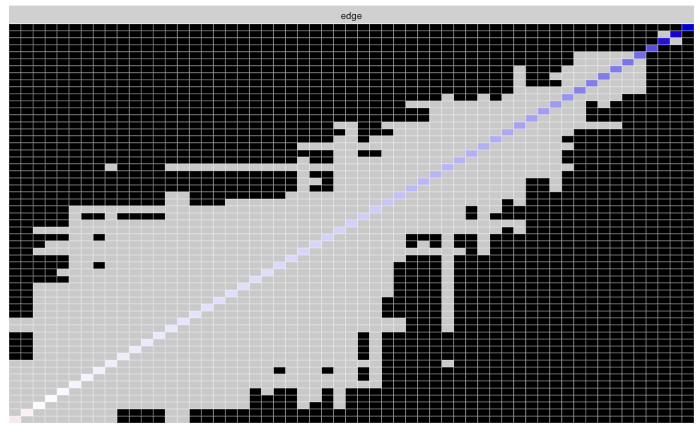


*Note.* Each unique node pair is represented on both the X and Y axis. Axis labels were removed for clarity. Gray boxes represent edges that do not significantly differ from one-another. Black boxes represent edges that do significantly differ from one another. Colored boxes correspond to the color of the edge in Figure 3.

Supplementary Figure 4. Bootstrapped Difference Tests Between Node Strength in the Estimated Network.


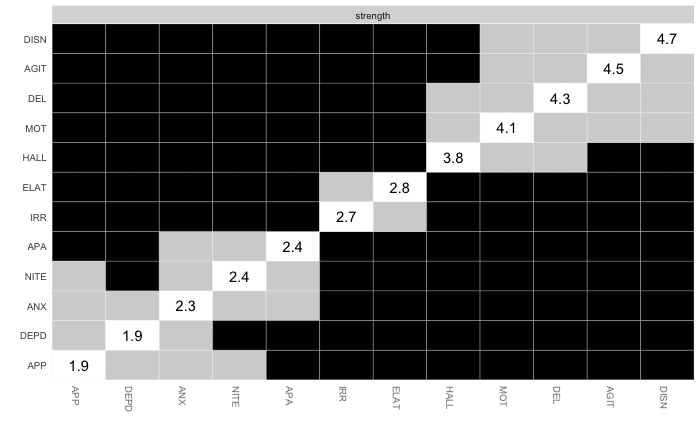


*Note.* Each node is represented once on the X axis and once on the Y axis. Gray boxes represent nodes that do not significantly differ from one-another. Black boxes represent nodes that do significantly differ from one another. White boxes show the value of node strength.

Supplementary Figure 5. Bootstrapped Difference Tests Between Node Expected Influence in the Estimated Network.


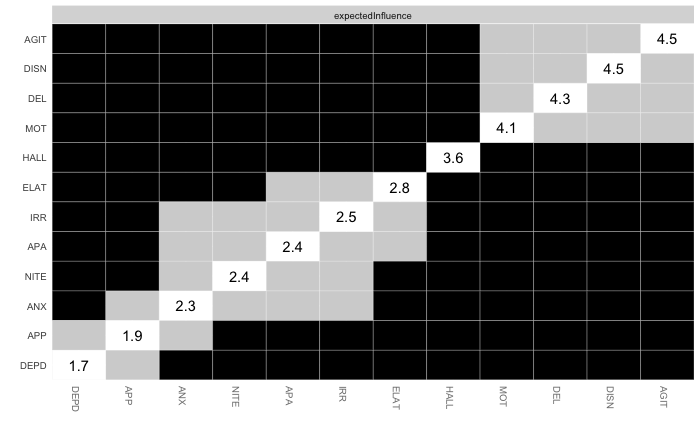


*Note.* Each node is represented once on the X axis and once on the Y axis. Gray boxes represent nodes that do not significantly differ from one-another. Black boxes represent nodes that do significantly differ from one another. White boxes show the value of node expected influence.
